# Less necessity of adjuvant S‐1 treatment in non‐monarchE‐eligible patients

**DOI:** 10.1002/cam4.6006

**Published:** 2023-05-10

**Authors:** Muhan Yu, Mamoru Takada, Hideyuki Yamada, Hiroshi Fujimoto, Junta Sakakibara, Hiroto Yamamoto, Takeshi Nagashima, Masayuki Ohtsuka

**Affiliations:** ^1^ Department of General Surgery, Graduate School of Medicine Chiba University Chiba Japan

**Keywords:** adjuvant therapy, CDK4/6, early breast cancer, inhibitor, luminal breast cancer, monarchE, POTENT, S‐1

## Abstract

**Background:**

In monarchE and Postoperative Therapy with Endocrine and TS‐1 (POTENT) trials, abemaciclib and S‐1 have, respectively, shown to be effective as adjuvant therapies for luminal breast cancer (BC), although whether patients who meet the criteria are at high risk of recurrence compared to non‐eligible patients is still unknown. Here, we investigated recurrence risk according to the criteria of each trial in Japanese patients.

**Methods:**

We reviewed the records of 992 patients who received surgery at Chiba University Hospital for stage I–III BC from January 2017 to May 2022 and selected 553 analytic cohort patients and retrospectively analyzed the relapse‐free survival of the patients as the primary endpoint. High‐recurrence risk was defined according to monarchE trial and POTENT trial.

**Results:**

The 5‐year RFS for monarchE cohort 1 and cohort 2 eligible patients were 77.78% and 89.33%, respectively, which were significantly lower than monarchE non‐eligible patients (98.31%; *p* < 0.0001). However, the 5‐year RFS rate for POTENT eligible patients (90.51%) was lower than for POTENT non‐eligible patients (98.75%; *p* = 0.0001); excluding those who met the monarchE criteria, the prognosis of POTENT eligible patients had no significant differences from the prognosis of patients with POTENT non‐eligible BC (*p* = 0.3100).

**Conclusion:**

MonarchE criteria accurately identify patients who are prone to relapse. Moreover, although POTENT criteria also suggested a reasonable capacity for recurrence prediction, there was no significant difference in recurrence between POTENT non‐eligible patients and the patients who were POTENT but not monarchE eligible. This might offer justification for reconsidering the use of S‐1 in monarchE non‐eligible patients.

## Graphical abstract

The research investigates the prognosis of monarchE or POTENT eligible patients and indicates the risk of recurrence in monarchE non‐eligible but POTENT eligible patients. MonarchE criteria accurately identifies patients at high risk of relapse but the relapse in the patients who only qualified for POTENT is statistically almost the same as that in POTENT non‐eligible patients.

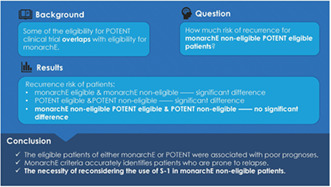

## 1 INTRODUCTION

Breast cancer (BC) is the most prevalently diagnosed carcinoma and the fifth major factor in women's cancer‐related death in Japan^1^, ^2^ and the second in the United States.^1^ There is a significant degree of molecular heterogeneity in BC, both across isolated tumors and within a single histological lesion of the tumor. Gene expression profiling has been used to define and characterize the major intrinsic molecular subtypes^3^ that differ in clinical behavior and response to treatment in BC practice. Subtype distinction is based on the simultaneous expression of three tumor markers: estrogen receptor (ER), progesterone receptor (PgR), and human epidermal growth factor receptor 2 (HER2). Characteristics of luminal BC include high hormone receptor (HR) expression and relatively low growth‐related gene expression. Luminal BC is the most diversified subtype and is prone to have a wide variety of progression and prognosis. Those with a good prognosis are sometimes grouped as luminal A and those with a poor prognosis as luminal B. Luminal B often has a high Ki‐67 proliferation index and is either HER2 positive or negative.

More than 90% of BCs are in the early stages (stages I, II, and III) when diagnosed.^4^ Early‐stage breast cancer (EBC) is usually treated with primary surgery, radiation therapy, chemotherapy, and adjuvant endocrine therapy (ET), all of which are considered standard treatments for patients with luminal EBC, depending on the risk of recurrence.^5^ However, nearly 30% of EBC patients experience recurrence^6^ of which most are distant metastasis,^7^ making the disease incurable. Studies have demonstrated that patients with specific clinical characteristics associated with high risk (including large initial tumor volume, widespread axillary lymph node [ALN] metastases, and high histologic grade) tend to have recurrence.^8^, ^9^, ^10^ In addition, high Ki‐67 expression in EBC patients is associated with a worse prognosis and has been previously demonstrated to be indicative of clinical outcome in EBC.^11^, ^12^ According to the criterion from the St Gallen International Expert Consensus, Ki‐67 20% was used to distinguish between low and high levels.^13^ Luminal BC with a Ki‐67 index larger than 20% and hyperproliferative disease, as evidenced by numerous multigene assays, has also been shown to be more likely to have a high risk of recurrence.^8^, ^14^, ^15^, ^16^, ^17^, ^18^ Furthermore, Harbeck et al. also reported that Ki‐67 ≥20% was found to be prognostic and may be utilized in conjunction with clinicopathological characteristics to identify individuals with a higher likelihood of recurrence.^19^ Identifying people with a high risk of recurrence can aid in therapy optimization and possibly prevent overtreating those who would not benefit from it.^20^, ^21^ As much as 20% of patients will relapse during the first 10 years and there has been limited improvement so far.^22^ In Japan, the 5‐year survival rate for all stages patients is 92.3%, whereas that for patients with distant metastases at the time of diagnosis is only 39.3%.^2^ In addition, patients who are women aged 20–75 years with histologically diagnosed stage I–IIIB invasive BC (intermediate to high risk of recurrence) should receive ET in the adjuvant setting.

Abemaciclib is a cyclin‐dependent kinase 4 and 6 inhibitor that could improve the survival of patients with luminal type advanced breast cancer when used with ET.^23^, ^24^, ^25^, ^26^ Research into the adjuvant setting of abemaciclib and ET is necessary. MonarchE (Clinicaltrials.gov registration: NCT03155997)^27^ trial examined the effects of adding abemaciclib to adjuvant ET in patients with high‐risk luminal subtype EBC. S‐1 has a relatively low occurrence of side effects, making it possible to be a good choice for chemotherapy.^28^ However, adjuvant chemotherapy of S‐1 has not been indicated. The purpose of Postoperative Therapy with Endocrine and TS‐1 (POTENT)^29^ clinical trial was to determine whether giving additional S‐1 reduces the relapse in women with luminal type EBC compared to giving normal postoperative ET. The results showed that patients who received S‐1 for 1 year prior to ET in the adjuvant setting were considerably less likely to relapse compared to those who were not treated by S‐1.^29^ These results provide a new adjuvant therapy option for luminal EBC patients with a high probability of recurrence: abemaciclib or S‐1.

Abemaciclib and S‐1 have demonstrated their value as adjuvant treatments for luminal BC in monarchE and POTENT clinical trials; however, how much the prognosis will be improved by S‐1 chemotherapy for the patients who did not meet monarchE but met POTENT criteria have not been reported yet. Moreover, it was found that in American patients, the risk of recurrence was three times higher in monarchE eligible patients than in non‐eligible patients.^30^ In addition, some of the eligibility for monarchE overlaps with eligibility for POTENT. However, for patients who meet POTENT but do not meet monarchE criteria, the amount of risk of recurrence and the benefit of additional S‐1 administration as adjuvant chemotherapy still remain unknown.

## 2 PATIENTS AND METHODS

### 2.1 Patients

**FIGURE 1:**
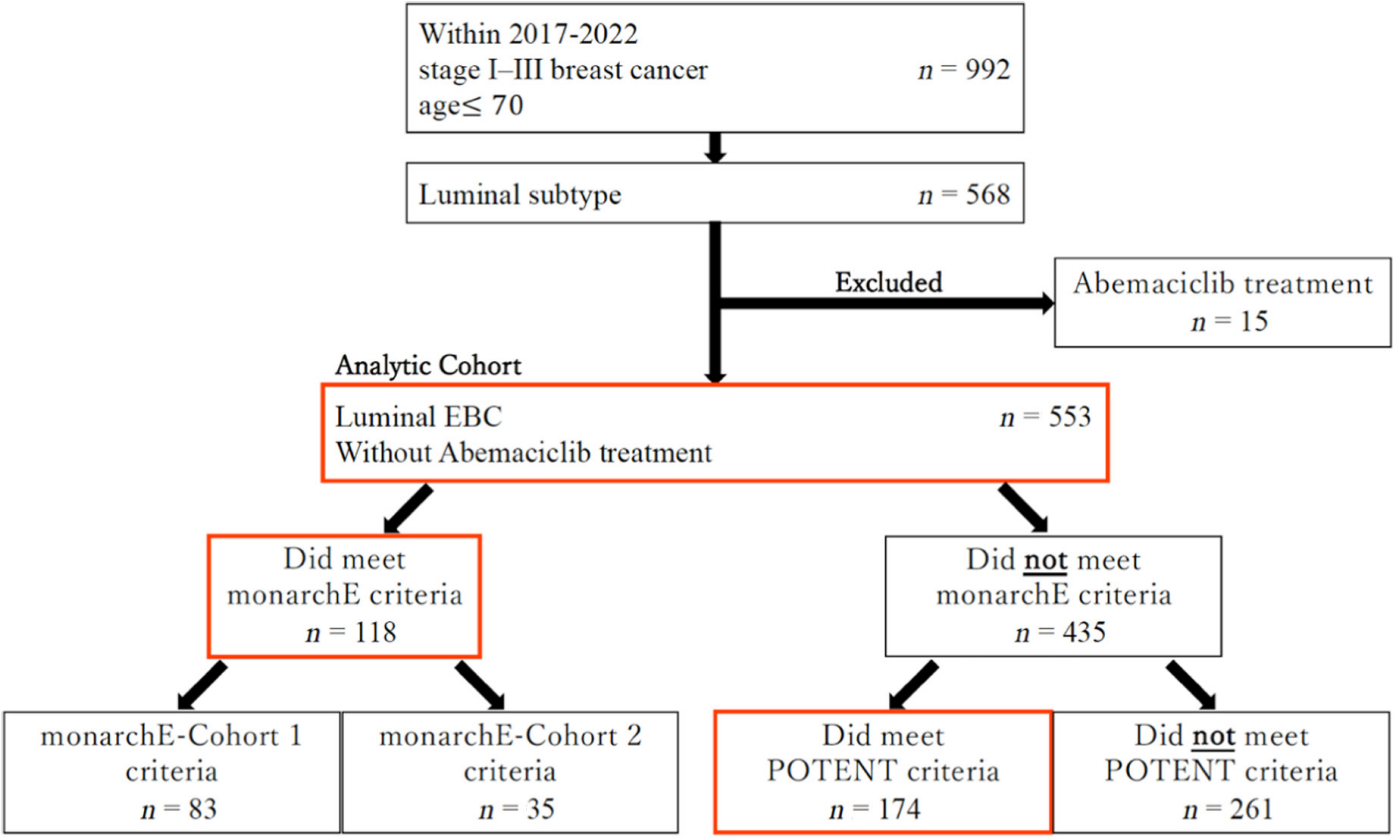
Flow chart of study selection process.

From January 2017 to May 2022, 992 patients under the age of 70 (at the time of surgery) who underwent surgery at Chiba University Hospital for stage I–III BC had their records reviewed. A total of 586 patients with stage I–III, hormone receptor‐positive and HER2‐negative BC were eligible; of these, 15 receiving abemaciclib treatment were excluded. Thus, 553 patients with luminal‐type BC were finally included (Figure 1).

The research related to human use has been complied with all the relevant national regulations, institutional policies and in accordance the tenets of the Helsinki Declaration, and has been approved by the Institutional Review Board of Chiba University Hospital (permission number: HK202302‐04, M10266). Due to the large analytic population of our study, it was approved by the Institutional Review Board of Chiba University Hospital that opt‐out could be used instead of written informed consent and all the patients included have not elected to opt out of this study.

### 2.2 High‐recurrence‐risk BC and the subgroup definitions

The pathology of all research participants was examined. The determination of ER, PgR, and HER2 status was conducted using immunohistochemistry (IHC), and fluorescent in situ hybridization (FISH) was also used as supplementary for HER2 2+ status. HER2 IHC 3+ or 2+/ FISH+ were regarded as indicators of HER2‐positive tumors. High‐recurrence‐risk was defined according to monarchE trial and POTENT trial clinicopathologic high‐risk criteria (Table S1). MonarchE criteria cohort 1 was defined as patients with HR+, HER2‐ stage I–III BC having either 4 positive ALNs, or 1–3 positive ALNs in combination with histological Grade 3, or 1–3 positive ALNs in combination with invasive tumor size larger than 5 cm. MonarchE criteria cohort 2 was defined as patients with one to three positive ALNs in combination with Ki‐67 larger than 20%. POTENT criteria included stage I–IIIB:

1. Adjuvant chemotherapy, or neither neoadjuvant nor prior adjuvant drug treatment.
  Eligible patients were positive for ALN metastasis by histology. Also, patients that were ALN metastasis‐negative were eligible if they have invasion diameter ≥3 cm, histological Grade 3, evident vascular invasion, or histological Grade 2 and invasion diameter ≥2 to <3 cm. Moreover, in lymph node metastasis‐negative cases with only histological Grade 2 or invasion diameter ≥2 to <3 cm, patients with high proliferation marker values in central assessment were eligible.
2. Neoadjuvant chemotherapy.
  Patients who were positive for ALN metastasis, or with residual primary or lymph node tumor, based on cytology or biopsy (needle biopsy or sentinel lymph node biopsy) performed before preoperative chemotherapy were eligible.
3. Neoadjuvant endocrine therapy.
  Patients who met the criteria in (1) according to histopathological examination of the tumor sample were also eligible. However, patients assessed to be ALN metastasis‐positive as a result of cytology or biopsy (needle biopsy or sentinel lymph node biopsy) performed before preoperative ET were also considered eligible regardless of the criteria in (1).

### 2.3 Statistical analysis

Fisher's exact test was used to assess differences in clinicopathological characteristics according to monarchE and POTENT eligible BC subgroups due to small sample sizes. The evaluated endpoint was recurrence‐free survival (RFS). RFS was defined as the period of time between diagnosis and BC recurrence. Within the recurrence analytic cohort, survival curves were estimated using Kaplan–Meier methods and compared with log‐rank tests. All tests assumed a 95% confidence interval (CI). Statistical significance was defined as a *p*‐value < 0.05. All analyses were conducted using Prism software version 9.0.0 (GraphPad Software Inc.).

## 3 RESULTS

### 3.1 Clinicopathological characteristics

We analyzed 553 eligible patients with HR+, HER2‐ stage I–III BC. The median follow‐up period was 41 months. There were 118 (21.34%) individuals with HR+, HER2‐ stage I–III BC who met monarchE criteria. 292 (52.80%) did and 261 (47.20%) did not meet POTENT criteria. Excluding the 118 patients who met monarchE criteria, there were 174 (31.46%) patients with stage I–IIIB luminal BC who met POTENT criteria and did not meet monarchE criteria. The clinicopathological characteristics, age, surgery type, T stage, N stage, histological grade, and chemotherapy of each criterion are listed in Table 1. MonarchE eligible BC was more likely to occur in patients at 40–55 years of age (45.76%), especially in the subgroup of 1–3 positive ALNs in combination with invasive tumor size larger than 5 cm (87.50%; Table S2), compared to those under 40 and over 55 years of age in the Japanese population. 61.86% of the patients defined by monarchE criteria received breast total mastectomy plus ALN dissection (Bt + Ax). Histological Grade II (61.02%) or 2–5 cm of tumor size (47.46%) were more commonly observed in monarchE eligible BC patients (Table S2). Among those who met monarchE criteria, 78.81% underwent chemotherapy (47.46% before surgery and 31.36% after surgery) and 75.42% underwent radiotherapy. In particular, among patients with four or more positive ALNs, 98.42% received chemotherapy (80.70% before surgery and 17.54% after surgery) and 87.72% received radiotherapy (Table S2). All of the monarchE eligible patients received ET. The recurrence of each subgroup of monarchE eligible patients is listed in Table S3.

**TABLE 1:** Clinicopathological characteristics according to the monarchE and Postoperative Therapy with Endocrine and TS‐1 (POTENT) criteria of patients.

Category	POTENT					*p*‐Value (Rec)	monarchE					*p*‐Value (Rec)	POTENT (monarchE excluded)					*p*‐Value (Rec)
	(*n* = 292)						(*n* = 118)						(*n* = 174)					
	No	%	Non Rec	Rec	Rec rate (%)		No	%	Non Rec	Rec	Rec rate (%)		No	%	Non Rec	Rec	Rec rate (%)	
Age at diagnosis						0.0264*												
≤40	31	10.62	26	5	16.13		19	16.10	15	4	21.05		12	6.90	11	1	8.33	
40–55	134	45.89	124	10	7.46		54	45.76	44	10	18.52		80	45.98	80	0	0	
>55	127	43.49	123	4	3.15		45	38.14	42	3	6.67		82	47.13	81	1	1.22	
Surgery type																		
Bt + SN	92	31.51	89	3	3.26		23	19.49	21	2	8.70		69	39.66	68	1	1.45	
Bp + SN	94	32.19	92	2	2.13		10	8.47	9	1	10.00		84	48.28	83	1	1.19	
Bt + Ax	86	29.45	72	14	16.28		73	61.86	59	14	19.18		13	7.47	13	0	0	
Bp + Ax	20	6.85	20	0	0		12	10.17	12	0	0		8	4.60	8	0	0	
Tumor size						<0.0001****						0.0562						0.0010***
<5 cm	260	89.04	250	10	3.85		92	77.97	82	10	10.87		168	96.55	168	0	0	
≥5 cm	32	10.96	23	9	28.13		26	22.03	19	7	26.92		6	3.45	4	2	33.33	
Histological grade						>0.9999						>0.9999						>0.9999
I or II	241	82.53	225	16	6.64		95	80.51	81	14	14.74		146	83.91	144	2	1.37	
III	51	17.47	48	3	5.88		23	19.49	20	3	13.04		28	16.09	28	0	0	
Chemotherapy						<0.0001****						0.1175						0.4079
Yes	133	45.55	116	17	12.78		93	78.81	77	16	17.20		40	22.99	39	1	2.50	
No	159	54.45	157	2	1.26		25	21.19	24	1	4.00		134	77.01	133	1	0.75	
Timing of chemotherapy						0.0341*						0.2634						>0.9999
Neoadjuvant	59	20.21	47	12	20.34		56	47.46	44	12	21.43		3	1.72	3	0	0	
Adjuvant	74	25.34	69	5	6.76		37	31.36	33	4	10.81		37	21.26	36	1	2.70	
Hormone therapy						>0.9999												>0.9999
Yes	289	98.97	270	19	6.57		118	100.00	101	17	14.41		171	98.28	169	2	1.17	
No	3	1.03	3	0	0		0	0	0	0	0		3	1.72	3	0	0	
Radiotherapy						0.4617						>0.9999						>0.9999
Yes	184	63.01	170	14	7.61		89	75.42	76	13	14.61		95	54.60	94	1	1.05	
No	108	36.99	103	5	4.63		29	24.58	25	4	13.79		79	45.40	78	1	1.27	

*Note*: Tumor size (invasive diameter) and the number of positive lymph nodes following primary surgery. However, in the patients who have received Neoadjuvant chemotherapy, the number of positive lymph nodes was conducted following computerized tomography scan at the time of diagnosis. Neoadjuvant, adjuvant, and no chemotherapy represent patients who received chemotherapy before surgery, after surgery, and did not receive chemotherapy, respectively. Yes and no for hormone therapy represents patients who did and did not receive hormone therapy, respectively. Yes and no from radiotherapy represents patients who received and did not receive radiotherapy, respectively.

Abbreviations: Ax, axillary lymph node dissection; Bp, breast partial mastectomy; Bt, breast total mastectomy; Rec, recurrence; SN, sentinel lymph node biopsy.

* *p* < 0.05, **p < 0.01, *** *p* < 0.005, **** *p* < 0.001.

Table 1 shows that in the Japanese patient population, a large proportion were over 40 years of age (89.38% for POTENT eligible patients and 93.11% for POTENT eligible monarchE non‐eligible patients) compared to those under 40 years of age. POTENT eligible patients at a young age (under 40 years old) had more frequent recurrence than patients over 40 years of age (*p* = 0.0264). Tumor size larger than 5 cm (compared with smaller than 5 cm) was an influential factor of recurrence regardless of the exclusion of monarchE eligible patients. Moreover, the patients who received chemotherapy had a higher recurrence rate than those who did not receive chemotherapy in POTENT criteria (*p* < 0.0001). Therefore, there are some patients who were low risk and have not relapsed even without receiving chemotherapy in POTENT criteria and chemotherapy failed to improve the prognosis for the POTENT eligible patients who had severe symptoms such as lymph node metastasis.

### 3.2 Comparison of patients with monarchE eligible and non‐eligible luminal‐type BC

**FIGURE 2:**
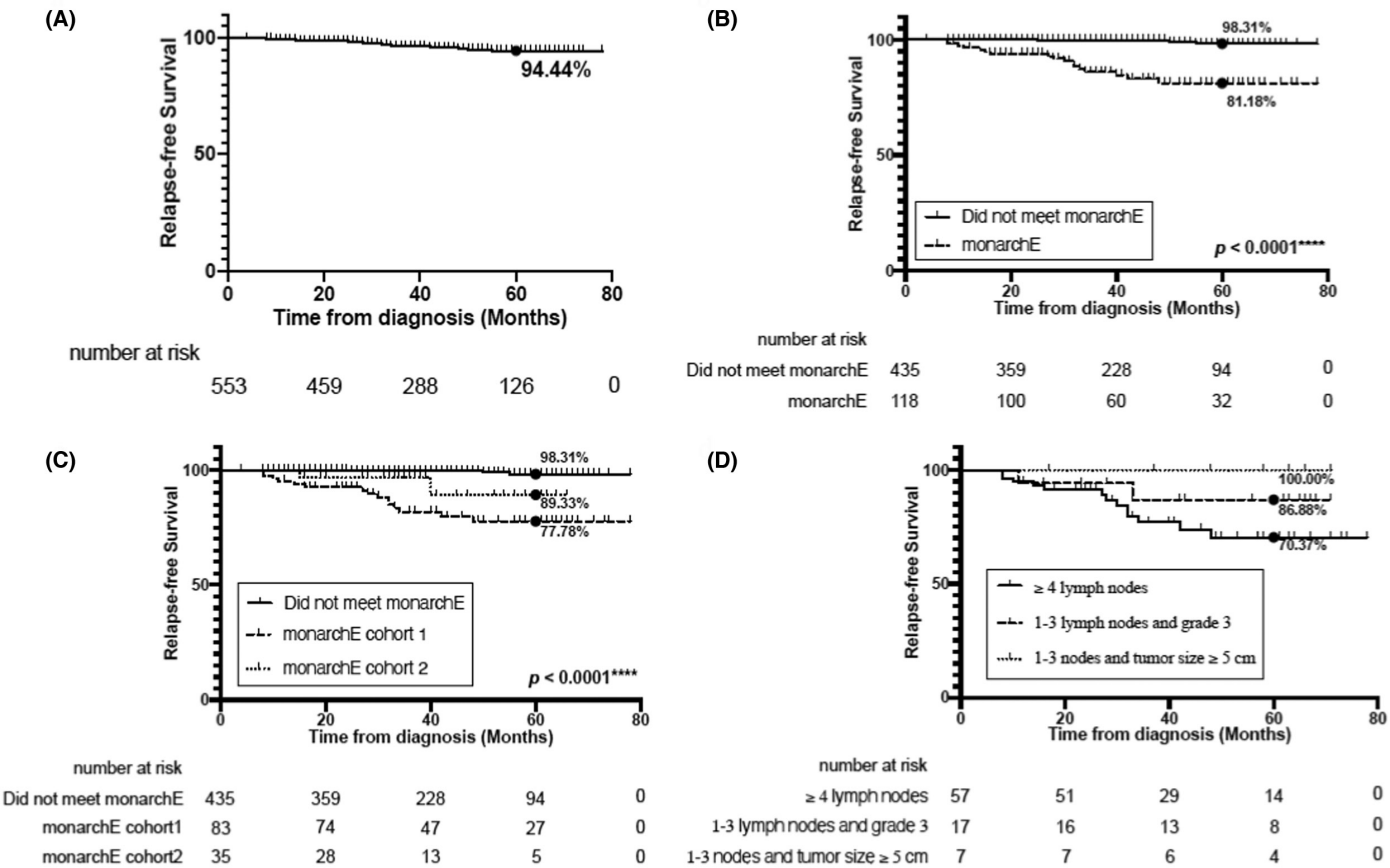
Kaplan–Meier survival curves of monarchE eligible patients and monarchE non‐eligible patients. (A) The 5‐year RFS rate for all luminal‐type breast cancer patients (94.44%). (B) Comparison of 5‐year RFS rate between the patients who did (81.18%) and did not meet (98.31%) monarchE criteria. (C) Comparison of 5‐year RFS rate between the patients who met monarchE cohort 1 (77.78%) and cohort 2 (89.33%) criteria and those who did not meet monarchE criteria. (D) Comparison of 5‐year RFS rate between the three subgroups of monarchE cohort 1: 4 or more positive axillary lymph nodes (ALNs) (70.37%), 1–3 positive ALNs with histological Grade 3 (86.88%), and 1–3 positive ALNs with tumor size ≥5 cm (100%).

A total of 20 (3.62%) stage I–III luminal‐type BC patients had recurrence within a short observation period of less than 5 years. The total 5‐year RFS rate for all luminal‐type BC patients was 94.44% (Figure 2A). Among the 553 patients with luminal‐type BC, 83 (15.01%) met monarchE cohort 1 criteria, 35 (6.33%) met cohort 2 criteria, and 435 (78.66%) did not meet clinicopathological criteria set in the monarchE clinical trial. Seventeen patients (14.41%) with monarchE eligible BC had recurrence, and all of the recurrences were distant metastases. The estimated 5‐year RFS rates for the patients who met monarchE (81.18%) were lower than that for the patients with monarchE non‐eligible BC (98.31%; *p* < 0.0001; Figure 2B), either in cohort 1 criteria (77.78%) or cohort 2 criteria (89.33%) (*p* < 0.0001, *p* = 0.0015; Figure 2C, Figure S4). There were no significant differences in recurrence between monarchE cohort 1 and cohort 2 criteria (*p* = 0.1434; Figure 2C). The 5‐year RFS rate for the subgroups of 4 or more positive ALNs, 1–3 positive ALNs with histological Grade 3, and 1–3 positive ALNs with tumor size ≥5 cm was 70.37%, 86.88%, and 100%, respectively (Figure 2D).

### 3.3 Comparison of patients with POTENT eligible and non‐eligible luminal‐type BC

**FIGURE 3:**
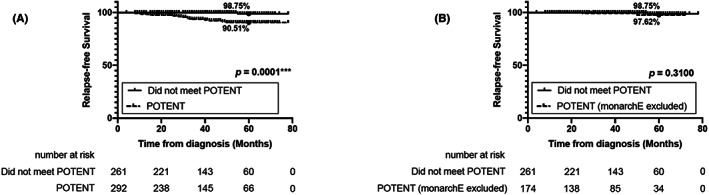
Kaplan–Meier survival curves of Postoperative Therapy with Endocrine and TS‐1 (POTENT) eligible patients and POTENT non‐eligible patients. (A) Comparison of 5‐year RFS rate between the patients who met POTENT (90.51%) and those who did not meet POTENT criteria (98.75%). (B) Comparison of 5‐year RFS rate between the patients who were only qualified for POTENT criteria (97.62%) and those who did not meet POTENT criteria.

Among 292 patients who met POTENT criteria, 19 patients (6.51%) had recurrences. Excluding the 118 patients who met monarchE criteria, 174 patients were disqualified from monarchE criteria but met POTENT criteria. Among those patients, 2 (1.15%) had relapsed. However, the 5‐year RFS rate for POTENT eligible BC patients (90.51%) was lower than that of the patients who did not meet POTENT criteria (98.75%; *p* = 0.0001; Figure 3A). When those who met the monarchE criteria were excluded, the survival of POTENT eligible patients (97.62%) had no significant differences from the patients with POTENT non‐eligible BC (*p* = 0.3100; Figure 3B).

## 4 DISCUSSION

### 4.1 The prognosis of Luminal‐type BC

Incidence‐based mortality and trends in women diagnosed with EBC from 2004 through 2018 were examined using the Surveillance, Epidemiology, and End Results database (Figure S1). Luminal BC subtype recurrence risk classifications, such as Oncotype DX and Prosigna‐PAM50, have been updated and put into practice with the development of treatments. Despite this, it is noteworthy that, from 2008 to 2016, EBC of luminal subtype has been reported to have little effect on improving survival probability^31^; similarly, in our analysis, EBC showed no improvement in survival over the past 15 years (AAC, 0.1%; 95% CI, 0.0%–0.2%). In luminal BC, clinical trials of monarchE and POTENT have demonstrated the benefit of abemaciclib and S‐1 as adjuvant therapy. However, some of the criteria of monarchE and POTENT are overlapping. Therefore, clinicians need to choose between abemaciclib and S‐1 for patients eligible for monarchE. Besides, there is no evidence yet on whether S‐1 should be used for patients who are eligible for POTENT but not for monarchE. Our results suggest that monarchE clearly and sensitively detects relapse‐prone patients and that most patients with relapse among those with POTENT criteria are eligible for monarchE. This might provide evidence for reconsidering the use of S‐1 in non‐eligible patients of monarchE. Nevertheless, the results of this study may be skewed due to the bias in the type of patients and the fact that it is a single university hospital retrospective study with a short and not absolutely rigorous follow‐up period. Therefore, further reporting would be required in the future.

### 4.2 Definition of high‐risk BC in luminal‐type BC

An important concept in the treatment of EBC is risk‐based adjuvant therapy. While the multigene assays described above have been developed for the most common luminal type of BC, it is the clinicopathological factors that all physicians refer to when considering treatment strategies. The combination of clinicopathological factors is used to select adjuvant therapy for luminal BC, and although it is necessary to understand the many reports on the weighting of these factors, the evidence is not always sufficient to make a more correct choice.

The effect of Prosigna®‐PAM50^32^ for long‐term outcome prediction was assessed in a population‐based study from the Danish Breast Cancer Group database of patients with luminal EBC at 50 years of age and met at least one of the risk criteria: tumor size >20 mm, ductal histology with malignancy Grade 2 or 3, or 1–3 positive ALNs. Tumors diagnosed as luminal B with PAM50 had a considerably worse prognosis than luminal A in both lobular (hazard ratio [HR]. 1.89; 95% CI, 1.03–3.45%; *p* = 0.04) and ductal (HR. 3.18; 95% CI, 2.29–4.43%; *p* < 0.0001) cancers.^32^ Although PAM50 has been shown to be useful in such ways in several studies (i.e., CALGB9741,^33^ GEICAM9906,^34^ TransATAC,^35^ ABCSG‐08,^36^ MA.5,^37^ and MA.12^38^ trials), it has yet to be widely used in actual clinical practice.

Nelson et al.^39^ reported that histological Grade 3 (compared with Grade 1) was the most influential on mortality (HR. 3.61; 95% CI, 3.27, 3.98; *p* < 0.0001). However, such findings have not been observed in our study. We supposed that there was a discrepancy attributed to the difference in the definition of histological grade and the existence of a special type in which it is difficult to describe the histological grade.

According to Pan et al.^40^ distant recurrence was highly associated with tumor size and positive lymph nodes at the time of diagnosis. While histological grade and Ki‐67, which often have a high correlation with each other, were only moderately effective independent predictors of distant recurrence; conversely, the PgR and HER2 did not display predicted values.

The monarchE trial enrolled patients with four or more positive ALNs or one to three positive ALNs with histological Grade 3 tumor or tumor size ≥5 cm in cohort 1, as well as patients with one to three positive nodes and a median high Ki‐67 index (>20%) in cohort 2. However, it has not yet been confirmed whether patients who meet the criteria of monarchE trial are at a high risk of recurrence compared to those monarchE non‐eligible patients in the Japanese community. Our study suggested that patients who met monarchE criteria, either cohort 1 or cohort 2, had worse prognosis than patients who did not meet the clinicopathological high‐risk criteria set in the monarchE, although abemaciclib adjuvant therapy to cohort 2 eligible patients has not been approved in Japan yet. Johnston et al.^41^ reported that there were no significant differences in invasive disease‐free survival and distant RFS between an abemaciclib plus ET group and an ET alone group in cohort 2. This was speculated to be due to the relatively low risk of recurrence in cohort 2 compared to cohort 1. In contrast, abemaciclib plus ET reduced the risk of recurrence compared to endocrine alone therapy in cohort 1 eligible patients.^41^ Moreover, Ki‐67 has been difficult to apply in clinical practice because of substantial interobserver heterogeneity, and there is currently no accepted threshold for defining the high and low status of Ki‐67.^42^ The value of the Ki‐67 threshold of ≥20% has been examined in the monarchE^27^ trial. Patients in cohort 1 with tumors that were Ki‐67 high had a greater chance of experiencing relapse than patients with tumors that were Ki‐67 low.^19^ These data support that high Ki‐67 status (≥20%) is an indicator with prognostic potential and may be used in conjunction with clinicopathologic factors in picking out high‐recurrence risk patients. However, our results indicated that there is no statistical difference in recurrence between Ki‐67 high (≥20%) and Ki‐67 low (<20%) patients in the monarchE cohort 1 (Figure S2, *p* = 0.7464). This may be attributed to differences in Ki‐67 determination methods, differences in patient backgrounds, and differences in chemotherapy regimens used in practice, such as higher dosage regimens selected for patients with higher Ki67 levels. Further investigation is needed on the clinical importance and application of Ki67 in luminal EBC.

The POTENT trial^29^ established criteria of intermediate to high risk of recurrence in BC. However, according to our results, although the recurrence for patients who met POTENT criteria was higher than the patients with POTENT non‐eligible BC, there were no substantial differences in recurrence between the patients who did not meet POTENT criteria and POTENT eligible patients who were not qualified for the monarchE criteria. In our study, 52.80% of patients meet POTENT criteria and this is the first report that implies the percentage of patients who met POTENT criteria in operable luminal BC and indicates the difference in recurrence of POTENT non‐eligible patients and patients who are not eligible for abemaciclib (monarchE) treatment but could be treated with S‐1 (POTENT). Our results indicate that the benefit of S‐1 therapy for POTENT eligible monarchE non‐eligible patients might be limited, as the relapse in this group is statistically almost the same as that in POTENT non‐eligible patients. However, it should be noted that 22.99% of POTENT eligible monarchE non‐eligible patients received chemotherapy.

It is reported that patients with ER‐positive and HER2‐negative disease, as well as intermediate to high risk (negative to positive for ALN metastasis), experienced absolute improvement of about 7%–8% in 5‐year iDFS with the addition of S‐1 to standard ET, while improvement was minimal in those at low risk. Besides, S‐1 was more effective in intermediate‐risk than high‐risk patients in the POTENT trial.^29^ In our results, POTENT high‐risk patients, 64.6% of whom have received chemotherapy, had a significantly higher risk of recurrence than POTENT intermediate‐risk patients, 20% of whom have received chemotherapy (Figure S3, *p* = 0.0018). Lymph node metastasis is a significant influential factor for relapse in both monarchE and POTENT eligible patients. Thus, it might be beneficial for POTENT high‐risk patients to receive additional treatment by S‐1 or abemaciclib, but not for POTENT intermediate patients. Although the results of the POTENT trial did not indicate the percentage of chemotherapy treatment in the high‐ and intermediate‐risk groups, the treatment history may have an influencing factor according to our data. Current evidence suggests that it is difficult to determine whether to add chemotherapy in patients at intermediate risk of recurrence. Among the patients who did not meet monarchE but did meet POTENT, 22.99% received chemotherapy. The fact that the risk of recurrence is similar for patients in our study between those who did not meet monarchE but did meet POTENT and those who met neither POTENT nor monarchE may indicate that we are correctly selecting patients who should receive chemotherapy out of the former group who are at higher risk.

In our study, patients who met the criteria of either monarchE or POTENT were associated with poor prognoses. The criteria set in each study are considered to be sufficiently appropriate. However, the relapse risk of patients who did not meet monarchE but did meet POTENT criteria was the same as patients who did not meet POTENT criteria and might not need adjuvant S‐1 therapy in our study. In other words, our results showed that monarchE criteria may be the more effective prognostic criteria than POTENT criteria in patients receiving adjuvant ET other than abemaciclib and S‐1. Although POTENT criteria also indicated a certain ability to predict recurrence, monarchE criteria performed much more accurately to identify patients at high risk and low risk of relapse.

While our results may clarify the validity of the monarchE criteria for the requirement of additional abemaciclib therapy, they may also allow us to consider the conflicting results of the PALLAS and PENELOPE B trials and the monarchE trial from a different perspective than that of drug potential.^43^, ^44^ Although there have been no analyses of the selection criteria for the adjuvant setting of the Palbociclib trial and no real clinical studies reports on the criteria, this may be an important future analysis for identifying the true high‐risk group for recurrence in luminal EBC.

Our research might assist in identifying individuals with high residual recurrence risk who would benefit from lengthier adjuvant chemotherapy regimens and enrollment in studies examining new medicines. In addition, through improvements in, for example, Ki‐67 evaluation methods and histological grading, and by combining liquid biopsy and comprehensive genomic profiling, including ctDNA, this recurrence prediction criteria may be optimized further to achieve even higher accuracy.

## AUTHOR CONTRIBUTIONS

**Muhan Yu:** Investigation (equal); writing – original draft (equal); writing – review and editing (equal). **Mamoru Takada:** Conceptualization (lead); funding acquisition (lead); investigation (lead); project administration (lead); writing – original draft (lead); writing – review and editing (lead). **Hideyuki Yamada:** Project administration (supporting); resources (supporting); writing – review and editing (supporting). **Hiroshi Fujimoto:** Project administration (supporting); resources (supporting); writing – review and editing (supporting). **Junta Sakakibara:** Project administration (supporting); resources (supporting); writing – review and editing (supporting). **Hiroto Yamamoto:** Project administration (supporting); resources (supporting); writing – review and editing (supporting). **Takeshi Nagashima:** Project administration (supporting); supervision (supporting). **Masayuki Ohtsuka:** Project administration (supporting); supervision (supporting).

## FUNDING INFORMATION

This work was supported by JSPS KAKENHI Grant number 21K08638.

## CONFLICT OF INTEREST STATEMENT

None.

## Supporting information


Data S1.

## Data Availability

N/A.
