# Supplementary material for: Less necessity of adjuvant S‐1 treatment in non‐monarchE‐eligible patients
Source: Cancer Med. 2023 May 10;12(12):13193–203. doi: 10.1002/cam4.6006 (PMC10315737; doi:10.1002/cam4.6006)

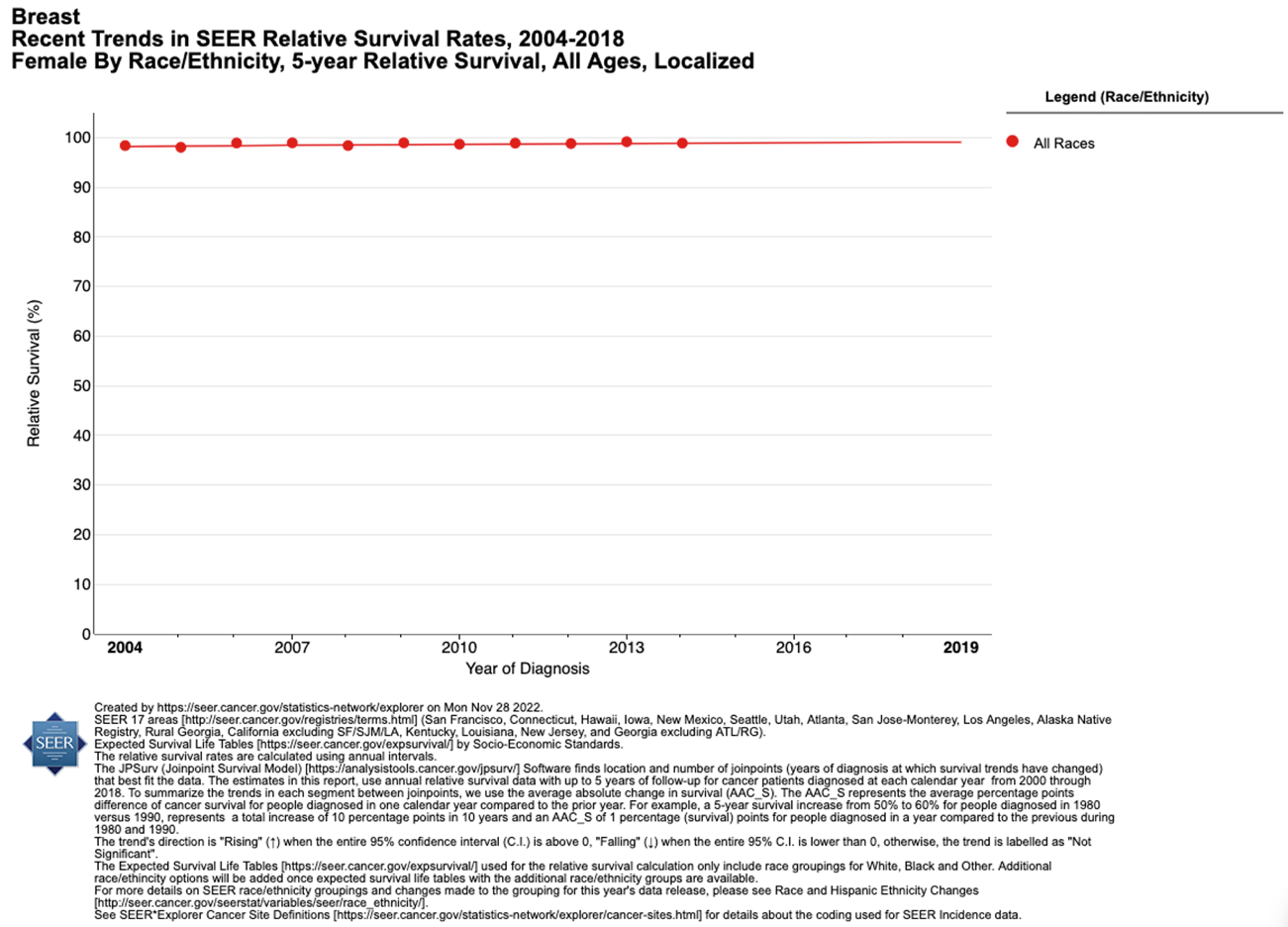


Supplementary Figure 1 Incidence-based 5-year relative survival rates and trends among EBC female patients of all ages from 2004 through 2018 according to SEER database.


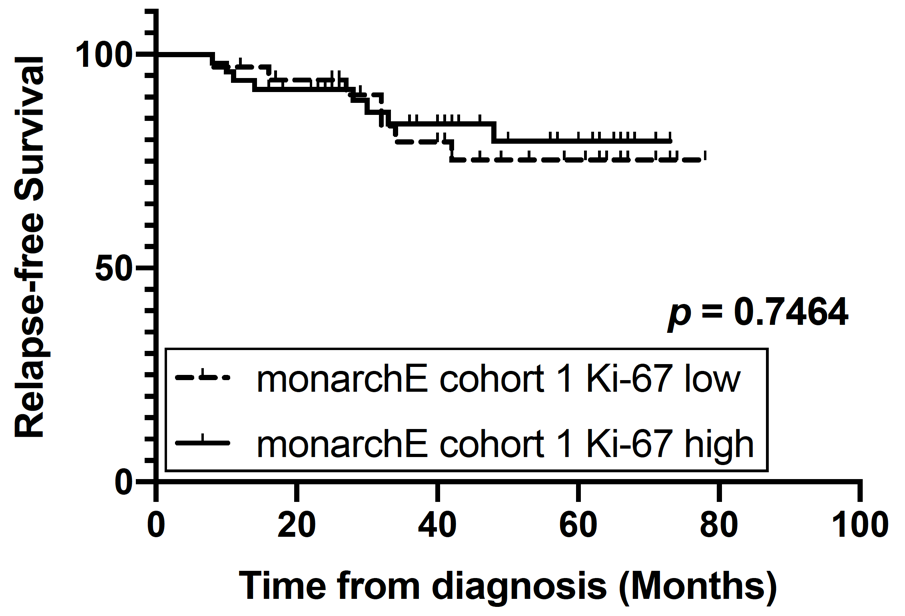


Supplementary Figure 2 Kaplan-Meier survival curves of monarchE cohort 1 Ki-67 low and Ki-67 high patients

Comparison of 5-year RFS rate between monarchE cohort 1 Ki-67 low and Ki-67 high patients.


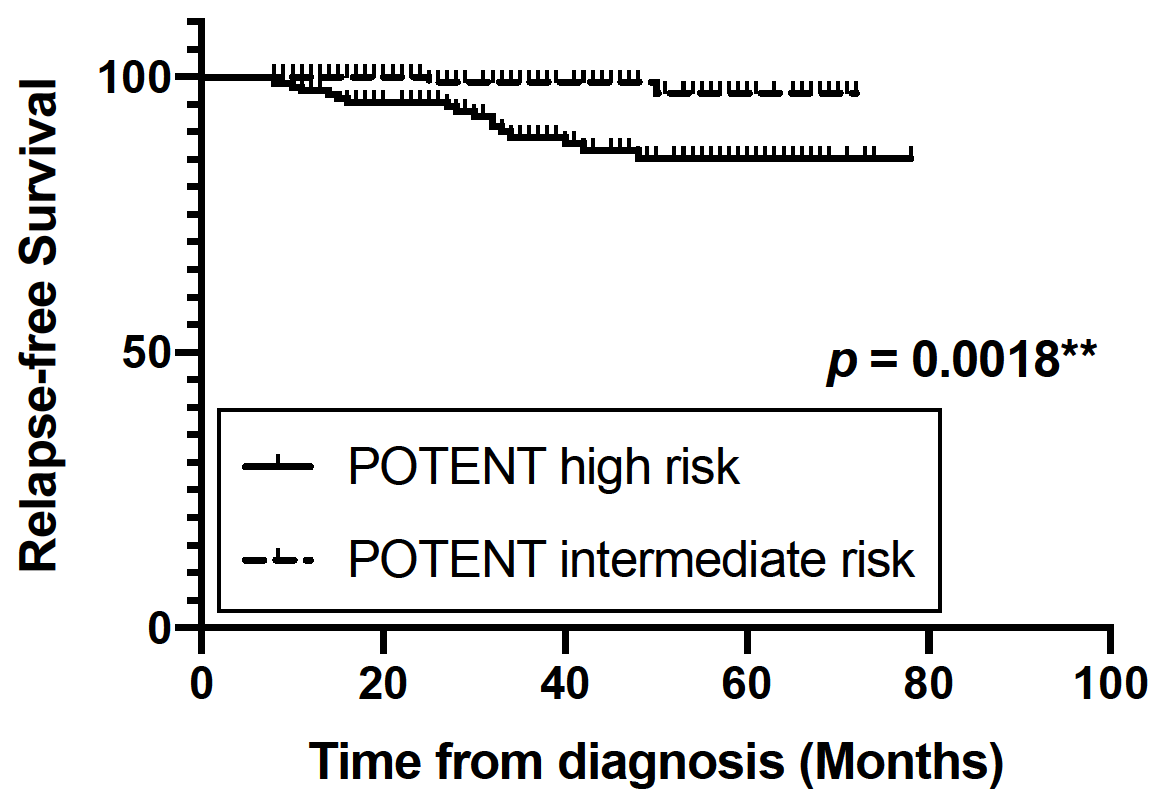


Supplementary Figure 3 Kaplan-Meier survival curves of POTENT high risk and intermediate risk patients

Comparison of 5-year RFS rate between POTENT high-risk patients and intermediate-risk patients.


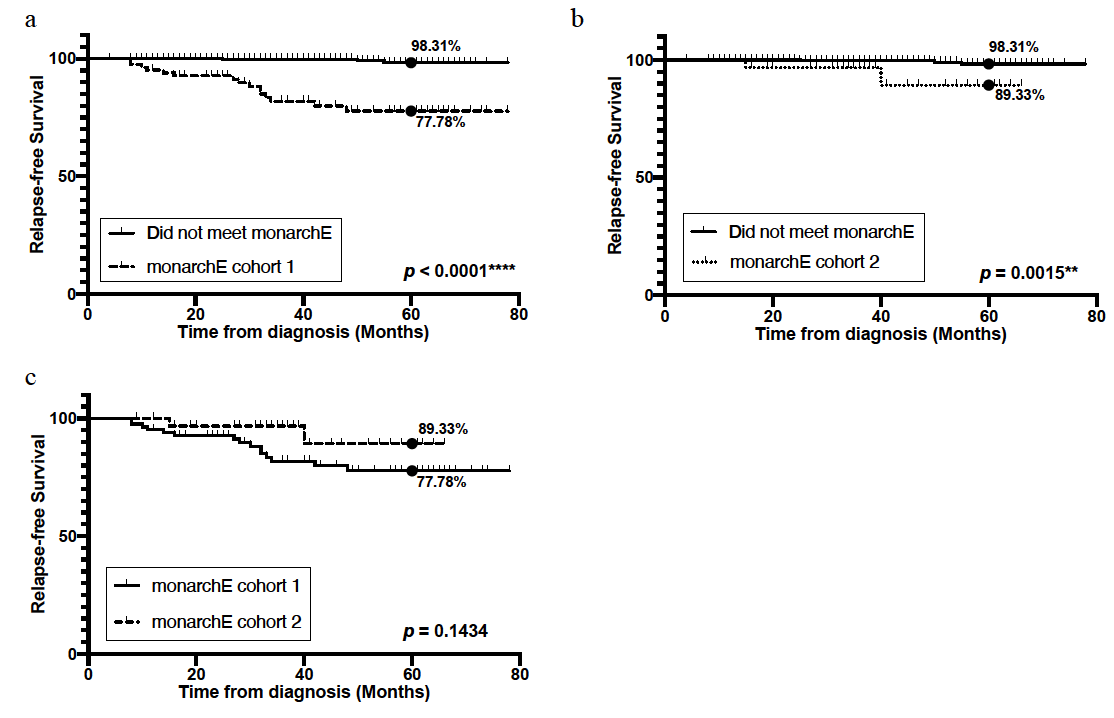


Supplementary Figure 4 Kaplan-Meier survival curves of monarchE eligible and monarchE non-eligible patients

1. Comparison of 5-year RFS rate between the patients who met monarchE cohort 1 criteria and those who did not meet monarchE criteria.
2. Comparison of 5-year RFS rate between the patients who met monarchE cohort 2 criteria and those who did not meet monarchE criteria.
3. Comparison of 5-year RFS rate between the monarchE cohort 1 and cohort 2 patients.

Supplementary Table 1 Summary table of monarchE and POTENT criteria


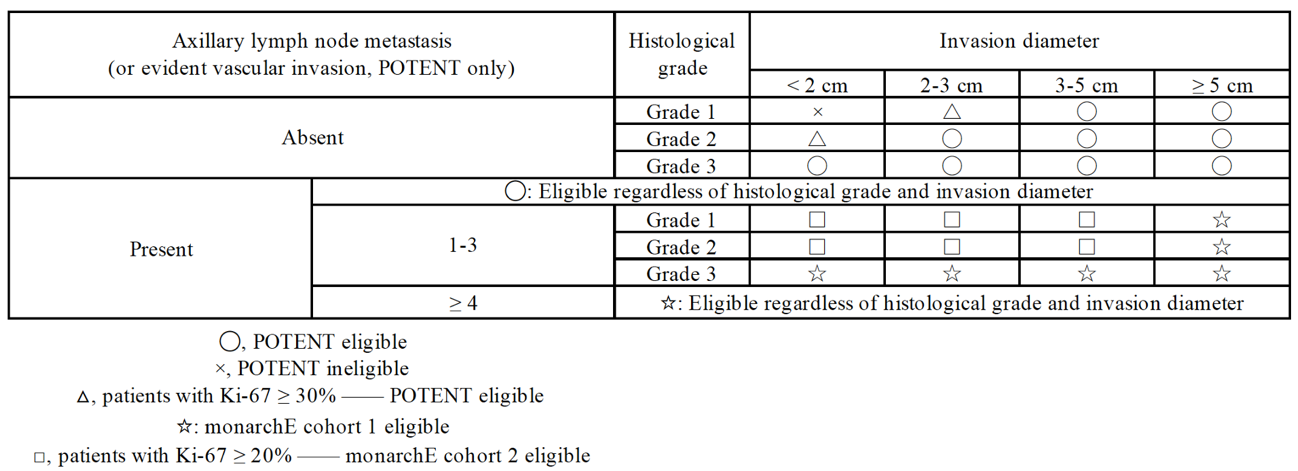


Supplementary Table 2 Clinicopathological characteristics according to the subgroups of monarchE eligible breast cancer (n=118)


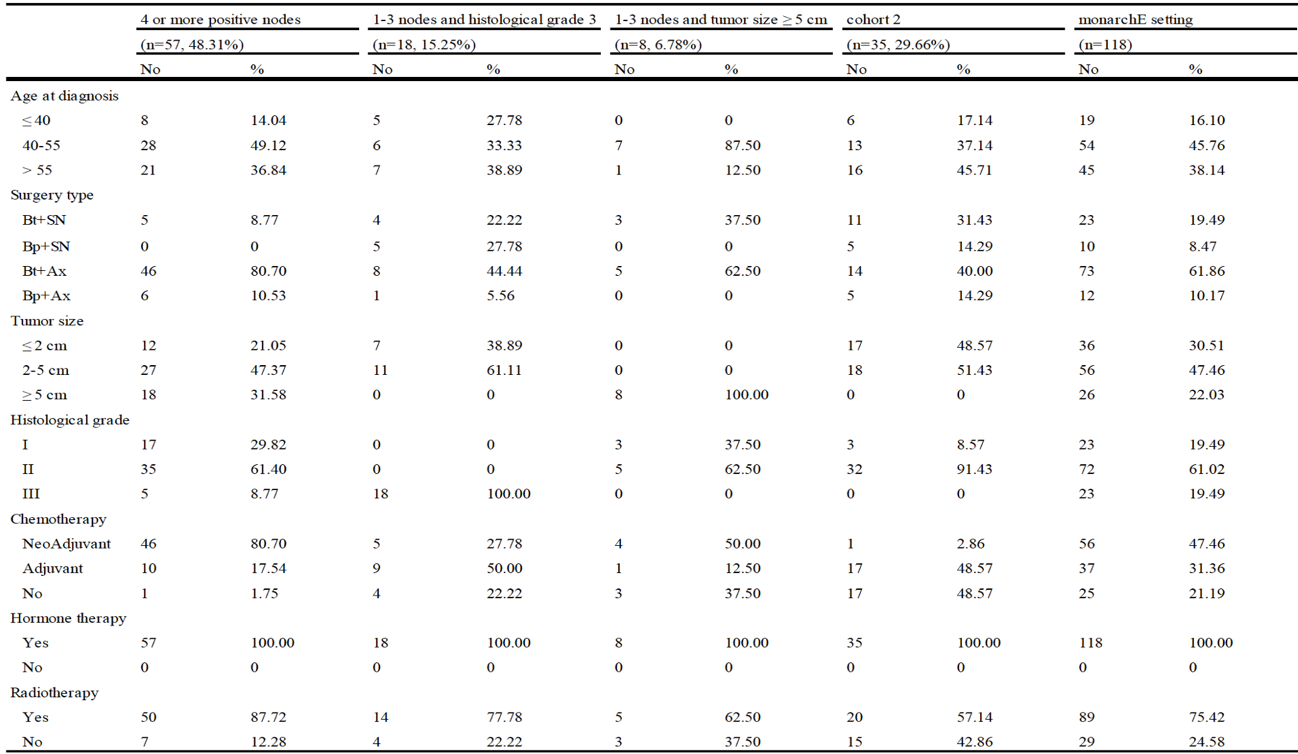


Tumor size (invasive diameter) and the number of positive lymph nodes following primary surgery. However, in the patients who have received Neoadjuvant chemotherapy, the number of positive lymph nodes was conducted following computerized tomography (CT) scan at the time of diagnosis.

Neoadjuvant, adjuvant, and no chemotherapy represent patients who received chemotherapy before surgery, after surgery, and did not receive chemotherapy, respectively.

Yes and no for hormone therapy represents patients who did and did not receive hormone therapy, respectively.

Yes and no from radiotherapy represents patients who received and did not receive radiotherapy, respectively.

Abbreviations:

Bp= breast partial mastectomy

Bt= breast total mastectomy

SN= sentinel lymph node biopsy

Ax= axillary lymph node dissection

Supplementary Table 3 Clinicopathological characteristics according to the recurrence (Rec) of subgroups of monarchE eligible breast cancer (n=118)


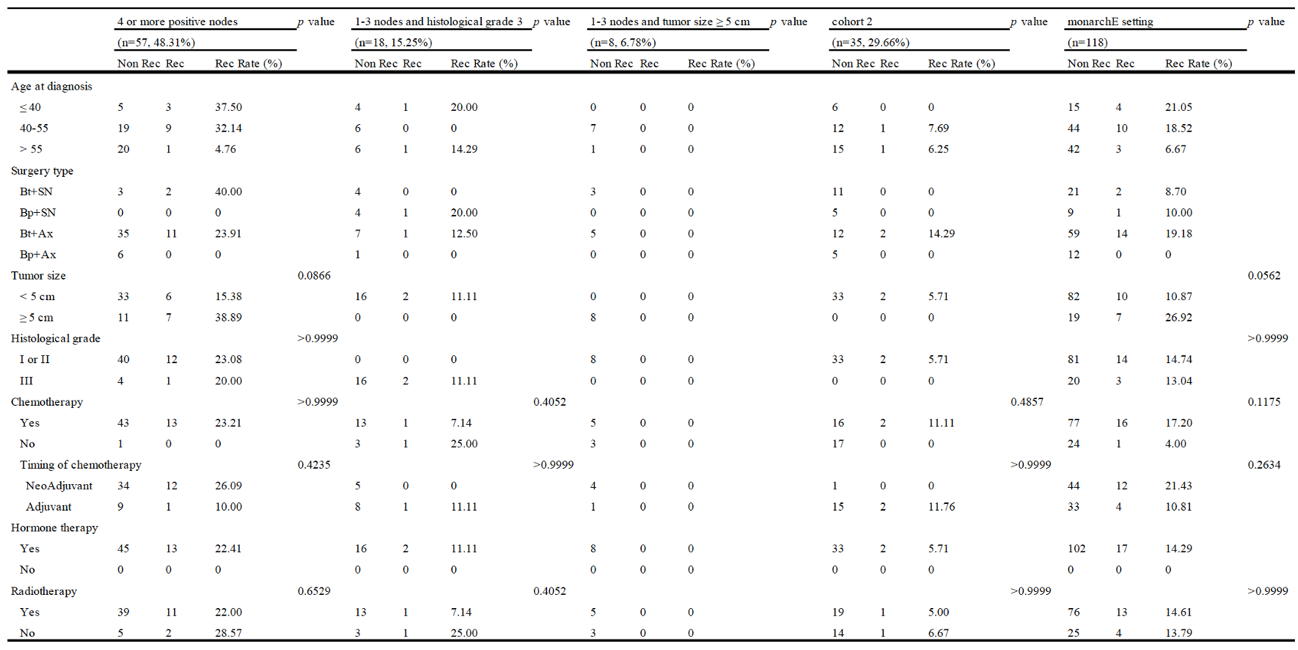

Supplement: Supplementary file 1 — Data S1. [file CAM4-12-13193-s001.docx]
